# Dynamics of the human skin mediator lipidome in response to dietary ω-3 fatty acid supplementation

**DOI:** 10.1096/fj.201901501R

**Published:** 2019-10-29

**Authors:** Alexandra C. Kendall, Suzanne M. Pilkington, Sharon A. Murphy, Francesco Del Carratore, Anggit L. Sunarwidhi, Magdalena Kiezel-Tsugunova, Paula Urquhart, Rachel E. B. Watson, Rainer Breitling, Lesley E. Rhodes, Anna Nicolaou

**Affiliations:** * Division of Pharmacy and Optometry, Laboratory for Lipidomics and Lipid Biology, School of Heath Sciences, Faculty of Biology, Medicine and Health, The University of Manchester, Manchester, United Kingdom;; † Division of Musculoskeletal and Dermatological Sciences, School of Biological Sciences, Faculty of Biology, Medicine, and Health, The University of Manchester, Manchester, United Kingdom;; ‡ Salford Royal National Health Service (NHS) Foundation Trust, Centre for Dermatology Research, Manchester Academic Health Science Centre, The University of Manchester, Manchester, United Kingdom;; § School of Chemistry, Faculty of Science and Engineering, The University of Manchester, Manchester, United Kingdom;; ¶ Lydia Becker Institute of Immunology and Inflammation, Faculty of Biology, Medicine, and Health, The University of Manchester, Manchester, United Kingdom;; ‖ National Institute of Health Research Manchester Biomedical Research Centre, Manchester University National Health Service (NHS) Foundation Trust, Manchester Academic Health Science Centre, Manchester, United Kingdom

**Keywords:** eicosapentaenoic acid, docosahexaenoic acid, inflammation, lipidomics, mass spectrometry

## Abstract

Nutritional supplementation with fish oil or ω-3 (n-3) polyunsaturated fatty acids (PUFAs) has potential benefits for skin inflammation. Although the differential metabolism of the main n-3PUFA eicosapentaenoic acid (EPA) and docosahexaenoic acid (DHA) could lead to distinct activities, there are no clinical studies comparing their relative efficacy in human skin. Following a 10-wk oral supplementation of healthy volunteers and using mass spectrometry-based lipidomics, we found that n-3PUFA mainly affected the epidermal mediator lipidome. EPA was more efficient than DHA in reducing production of arachidonic acid–derived lipids, and both n-3PUFA lowered *N*-acyl ethanolamines. In UV radiation–challenged skin (3 times the minimum erythemal dose), EPA attenuated the production of proinflammatory lipids, whereas DHA abrogated the migration of Langerhans cells, as assessed by immunohistochemistry. Interestingly, n-3PUFA increased the infiltration of CD4^+^ and CD8^+^ T cells but did not alter the erythemal response, either the sunburn threshold or the resolution of erythema, as assessed by spectrophotometric hemoglobin index readings. As EPA and DHA differentially impact cutaneous inflammation through changes in the network of epidermal lipids and dendritic and infiltrating immune cells, they should be considered separately when designing interventions for cutaneous disease.—Kendall, A. C., Pilkington, S. M., Murphy, S. A., Del Carratore, F., Sunarwidhi, A. L., Kiezel-Tsugunova, M., Urquhart, P., Watson, R. E. B., Breitling, R., Rhodes, L. E., Nicolaou, A. Dynamics of the human skin mediator lipidome in response to dietary ω-3 fatty acid supplementation.

Studies following nutritional supplementation with either fish oil or ω-3 (n-3)polyunsaturated fatty acid (PUFA) formulations suggest their potential benefits in a number of inflammatory skin conditions, including atopic dermatitis, psoriasis, lupus erythematosus, UV radiation (UVR)-induced inflammation, and wound healing (1–5). The bioactivity of n-3PUFA is attributed to production of lipid mediators, perturbation of membrane lipid composition, and altered gene and protein expression (5–9). These events activate signaling pathways and modify the production of chemotactic lipid or protein mediators that change the skin microenvironment, with impact on the profile and activation of resident and infiltrating immune cells (10–12).

Although the principal n-3PUFA eicosapentaenoic acid (EPA) and docosahexaenoic acid (DHA) share anti-inflammatory properties and tend to be studied together as components of fish oil, they have clear differences in terms of their metabolism and function. Whereas EPA can generate eicosanoids through its metabolism by cyclooxygenase (COX), lipoxygenase (LOX), and cytochrome P450 (CYP450) isoforms, DHA is mainly metabolized by LOX and CYP450 to form docosanoids (13–15). Transcellular metabolism of EPA and DHA generates proresolving lipid mediators of the resolvin (Rv), protectin (PD), and maresin classes, whereas both fatty acids are precursors of *N*-acyl ethanolamines (NAEs) that are related to the endocannabinoids (eCBs) (16–18). There is no consensus on whether eicosapentaenoyl ethanolamine (EPEA) and docosahexaenoyl ethanolamine (DHEA) actively bind to the cannabinoid receptors, making them true eCBs, but they exhibit biologic activities in various tissues, possibly through receptors such as transient receptor potential vanilloid 1 and peroxisome proliferator-activated receptors [reviewed in Meijerink *et al*. (19)].

Studies comparing the relative efficacy of EPA and DHA have shown that they appear to differently affect production of cutaneous ceramides in human skin *ex vivo*, whereas DHA is reportedly more efficient than EPA at reducing circulating inflammatory markers, inhibiting gene expression in Jurkat cells, and modifying clustering of membrane lipid microdomains in T lymphocytes (20–23). In contrast, EPA, but not DHA, was shown to prevent systemic immunosuppression in rodents after UVR exposure (24). Although these studies suggest that the differential metabolism of EPA and DHA could lead to distinct activities in skin, only EPA has been studied for its effect on human cutaneous inflammation, and to our knowledge, there are no clinical studies of the skin testing DHA alone (25, 26).

Furthermore, most studies on skin lipids focus on the role of n-3PUFA in the epidermis and do not explore the significance of the dermal contribution. Epidermis and dermis have distinct cellular populations both in terms of resident skin cells and infiltrating immune cells, and the dermis plays a significant role in supporting the avascular epidermis physically, biochemically, and nutritionally. As epidermal keratinocytes rely upon systemic delivery of long chain PUFA (because of a lack of epidermal Δ5 and Δ6 desaturase activity), cutaneous lipid profiles are subject to systemic and nutritional influences (27). Consequently, it is important to appraise the impact of EPA and DHA independently on the mediator lipidome of these skin compartments, as such insight may reveal novel ways of strengthening the skin’s response against inflammatory challenges and, potentially, contribute to the treatment of cutaneous inflammatory diseases.

We have therefore explored the metabolism and individual roles of EPA and DHA in human epidermis and dermis following a 10-wk dietary supplementation with each n-3PUFA, together with a proinflammatory UVR challenge designed to stimulate production of bioactive lipid mediators. As well as following changes in the mediator lipidome, we assessed the expression of lipid metabolizing enzymes and monitored changes in cutaneous immune cells. These studies provided insights into the potential immunoregulatory effects of the 2 n-3PUFA supplements, whereas comparison of the cutaneous and circulating mediator lipidomes allowed us to further appraise the degree to which systemic levels of EPA and DHA reflect the local skin environment. Our findings reveal that EPA and DHA differentially influence the network of epidermal and dermal lipids and cellular components of innate and adaptive immunity, with implications for the skin’s response to inflammation and consequence for the design of interventions targeting cutaneous disease.

## MATERIALS AND METHODS

### Participants

Healthy male and female volunteers were recruited to either the EPA (*n* = 12) or the DHA (*n* = 9) oral supplementation study. Volunteers were eligible to take part if they were white Caucasian, aged 18–60 yr old to avoid age-related skin changes that occur above the age of 70, and of sun-reactive skin type I–III (28), with no preexisting skin conditions or other medical conditions that required the use of systemic medications. Participant ages were 21–58 yr (mean 41.5 yr) in the EPA study, and 21–53 yr (mean 32.3 yr) in the DHA study. Ethical approval was granted by the National Research Ethics Committee North West (approval 11/NW/0567) and the study was performed in accordance with the Declaration of Helsinki principles (revised Seoul 2008); all volunteers provided written informed consent.

### Study design and intervention

The studies were performed consecutively as open-label supplementation studies. The supplements were 1 g gelatin capsules containing either Incromega E7010 SR ethyl ester (∼70% EPA, 10% DHA) or Incromega E1070 SR ethyl ester (10% EPA, 70% DHA) (Croda Chemicals, Leek, United Kingdom). The fatty acid content of the capsules was confirmed in-house using gas chromatography (GC) with flame ionization detection (Supplemental Fig. S1). Volunteers were assigned to either the EPA or DHA intervention, and supplements were taken as 5 capsules daily with breakfast for 10 wk (corresponding to 3.5 g EPA or DHA/d). All volunteers provided fasting blood samples to assess supplement compliance and plasma lipid mediators. Additionally, small areas of photoprotected upper buttock skin were exposed to UVR to determine the sunburn threshold (29) and induce inflammation prior to skin sampling *via* punch biopsy. All tissue sampling and clinical procedures were performed both pre- and postsupplementation.

### Tissue sampling

Fasting blood samples (7 ml) were taken and centrifuged for 15 min at 2500 *g* to separate red blood cells (RBCs) and plasma fractions (EDTA, 1.8 mg/ml), which were collected and stored at −80°C until analysis. Skin biopsies were taken using 6-mm diameter Militex biopsy punches (Militex, York, PA, USA) following intradermal injection of lidocaine (2%) local anesthetic (Antigen Pharmaceuticals, Tipperary, Ireland). Samples were taken in duplicate from unexposed and UVR-exposed skin for each time point (24 and 72 h), prepared by formalin fixation, paraffin embedding, and snap-freezing in optimal cutting temperature compound (Cell-Path, Powys, United Kingdom) for histologic analyses, or snap-frozen for lipidomic analyses. Snap-frozen samples were stored at −80°C, and all samples were analyzed at completion of the human study.

### RBC fatty acid analysis

RBCs were defrosted on ice before extraction with chloroform:methanol (4 ml; 2:1, v/v) with 0.01% (w/v) butylated hydroxytoluene, fatty acids were converted to fatty acid methyl esters (FAME) *via* acid-catalyzed transesterification using boron trifluoride in methanol, and FAMEs were analyzed by GC with flame ionization, using heneicosanoic acid (21:0) as an internal standard, as previously described (30).

### Extraction and ultraperformance liquid chromatography with electrospray ionization and tandem MS analysis of eicosanoids and related species

Skin was divided into dermis and epidermis (on ice, by scalpel, with the aid of visual inspection at ×40 magnification) as previously described (17, 20). Skin (5–30 mg) and plasma samples (∼1 ml) were extracted using ice-cold methanol (15%, v/v), and 12-hydroxyeicosatetraenoic acid (HETE)-*d*_8_, prostaglandin (PG)B_2_-*d*_4_, 8 (9)-epoxy eicosatrienoic acid-*d*_11_, and 8,9-dihydroxyeicosatrienoic acid-*d*_11_ (20 ng each/sample; Cayman Chemicals, Ann Arbor, MI, USA) were used as internal standards. The extracts were semipurified using solid-phase extraction cartridges (C18-E; Phenomenex, Macclesfield, United Kingdom) to eliminate matrix effects. Ultraperformance liquid chromatography with electrospray ionization and tandem mass spectrometry (UPLC/ESI-MS/MS) analysis was performed on an Acquity UPLC pump (Waters, Wilmslow, United Kingdom) coupled to an electrospray ionization triple quadrupole mass spectrometer (Xevo TQ-S; Waters) as previously described (17, 31, 32). A detailed list of the multiple reaction monitoring transitions and collision energy settings is provided in Supplemental Table S1. Results are expressed as picograms per milligram protein (skin) or picograms per milliliter (plasma).

### Extraction and UPLC-MS/MS analysis of eCBs and NAEs

Skin (5–30 mg) and plasma (∼1 ml) samples were extracted using ice-cold 2:1 (v/v) chloroform/methanol, as previously described (17, 33). Arachidonoyl-EA-*d*_8_ and 2-arachidonoyl glycerol (AG)-*d*_8_ (20 and 40 ng per sample, respectively; Cayman Chemicals) were used as internal standards. Lipid extracts were analyzed by UPLC/ESI-MS/MS (a list of multiple reaction monitoring and collision energy settings is provided in Supplemental Table S2). Results are expressed as picograms per milligram protein (skin) or picograms per milliliter (plasma).

### Protein content

During lipid extractions, protein pellets were retained for analysis using a standard Protein Assay Kit (Bio-Rad, Hercules, CA, USA). Proteins were solubilized using 1 M NaOH and analyzed within the linear range of the assay, as previously described (15, 17).

### UVR skin exposure

UVR was administered using a Waldmann UV 236B unit housing 2 Waldman fluorescent broadband UVR lamps (280–400 nm; peak 313 nm; Herbert Waldmann, Germany). Presupplementation, a geometric series of 10 erythemally weighted UVR doses (8–80 mJ/cm^2^) was applied to the upper buttock of each volunteer, and the individual’s minimum erythemal dose (MED; sunburn threshold) was determined at 24 h postexposure. In order to provide a proinflammatory stimulus, two 1-cm-diameter sites on the upper buttock were exposed to 3 times the individual’s MED (3× MED) of UVR at 24 and 72 h prior to skin sampling. Following the supplement course, the MED test and the same individualized UVR challenges (3× MED) were repeated.

### Skin erythema assessment

The MED was defined as the lowest dose of UVR (millijoules per centimeter square) causing a visually perceptible erythema at 24 h after UVR exposure as previously described (29). Erythemal intensity was also measured using a spectrophotometer (CM600d; Konica Minolta Sensing Europe, Gothenburg, Sweden) to provide hemoglobin index readings. These readings were performed at 24 and 72 h after UVR exposure. Additionally, hemoglobin index readings were taken at sites exposed to 3× MED UVR at 24, 48, and 72 h.

### Immunohistochemical analysis

Formalin-fixed skin biopsies were embedded in paraffin wax, sectioned at 5 μm thickness, and stained for markers of neutrophil elastase, CD4, CD8 (T-cell markers), and COX-2, whereas snap-frozen skin samples were cryosectioned at 7-μm thickness and stained for 12-LOX, 15-LOX, *N*-acyl phosphatidylethanolamine–specific phospholipase D (NAPE-PLD), and CD207 (Langerin) for Langerhans cells (LCs). All washes were performed in tris-buffered saline. The primary antibodies used in this study were as follows. Neutrophil elastase: NP57, dilution 1:100 (Agilent Technologies, Santa Clara, CA, USA); CD4: 4B12, dilution 1:50 (Agilent Technologies); CD8: C8/144B, dilution 1:100 (Agilent Technologies); 12-LOX: rabbit anti-ALOX12, dilution 1:200 (MilliporeSigma, Burlington, MA, USA); 15-LOX: ab23691, dilution 1:100 (Abcam, Cambridge, MA, USA); COX-2: CX-294, dilution 1:25 (Agilent Technologies); NAPE-PLD: ab133181, dilution 1:100 (Abcam); LC (langerin): anti-CD207, dilution 1:200 (MilliporeSigma). The secondary antibodies were as follows: neutrophil elastase, CD4, COX-2, 12-LOX, 15-LOX, and NAPE-PLD: Vector ImmPress (Vector Laboratories, Burlingame, CA, USA); CD8: Dako Envision (Agilent Technologies); LC: AF594, dilution 1:200 (Abcam). Substrates and counterstains were as follows: CD8, CD4: diaminobenzidine and Mayers hemotoxylin (Agilent Technologies and MilliporeSigma); neutrophil elastase, COX-2: Vector SG with nuclear fast red (Vector Laboratories); 12-LOX, 15-LOX, NAPE-PLD: Nova Red and hemotoxylin (Vector Laboratories and MilliporeSigma); LC: DAPI (MilliporeSigma). Stained slides were imaged using a ×20/0.80 Plan Apo objective by the 3D Histech Panoramic 250 Flash II slide scanner in the Bioimaging facility (University of Manchester, Manchester, United Kingdom). Using the 3D Histech Pannoramic viewer software, leukocyte markers were analyzed by counting the number of positively stained cells in 3 high-power fields (HPFs) for neutrophils and CD8^+^ T cells (×200) and in 4 HPFs for CD4^+^ T cells (×400). Slides stained for LC markers were imaged using an Olympus BX53 fluorescence microscope and Olympus Cell Sens software; positively stained LCs were counted in 3 HPFs (×100). Enzyme staining was analyzed as % area of skin stained using ImageJ software (National Institutes of Health, Bethesda, MD, USA) (34). Cell counts and percentage area staining analysis were performed in 3 skin sections per condition.

### Statistical analysis

Lipid mediator data were subjected to univariate paired analyses, performed on log-transformed data. For each pairwise comparison, missing values were treated according to 2 simple heuristics: mediators missing in more than half of the samples were removed from the dataset and, when less than half of the values of a mediator were missing, these were substituted with the analytical limit of detection. Variance stabilization and normalization using the vsn R package (*https://bioconductor.org/packages/release/bioc/html/vsn.html*) was applied to all datasets (35). In order to identify differentially expressed metabolites, a 2-tailed, paired Student’s *t* test was used and *P* values were corrected for multiple testing using the Benjamini-Hochberg method to control false discovery rates at *P* = 0.05 (36). Furthermore, iterative group analysis (iGA) was applied to each pairwise comparison to identify global changes (37). For these analyses, the lipid mediators were clustered into 8 groups based on common features (Supplemental Table S3), and the enrichment score (PC) reflecting the global changes in the concentration of lipids in each group was estimated; the scores were corrected for multiple testing, and a PC value of <5 × 10^−4^ was considered significant. All data processing and statistical analyses were implemented in custom-made R scripts.

The subsequent analyses were performed using Prism v.7.00 (GraphPad Software, La Jolla, CA, USA). In detail, cellular infiltrate, enzyme expression, and hemoglobin index data were analyzed by 2-way repeated measures ANOVA followed by Tukey’s multiple comparison tests; changes in RBC fatty acid composition were analyzed by paired Student’s *t* tests; correlations between cutaneous and plasma lipids were analyzed using Spearman rank correlations; and for all these tests, a value of *P* < 0.05 was considered significant.

## RESULTS

### EPA and DHA increase RBC *n*-3PUFA levels confirming compliance and supplement bioavailability

All volunteers took the supplements without adverse effects, completed the study and were compliant, as assessed by GC of FAME analysis of RBC fatty acids (38) (Supplemental Fig. S2). Increased RBC levels of EPA (*P* = 0.001) and DHA (*P* < 0.0001) were observed postsupplementation with the respective fatty acids. Indeed, EPA supplementation raised the mean n-3 index from 4.86 to 7.67%, whereas DHA supplementation increased the mean n-3 index from 4.39 to 8.25% (39). Furthermore, EPA supplementation reduced RBC arachidonic acid (AA) content (*P* = 0.001), consistent with previous reports (40–42), and increased levels of the EPA elongation and desaturation products docosapentaenoic acid (*P* < 0.0001) and DHA (*P* = 0.025), although some of the observed increases in DHA could be attributed to the small amount of DHA included in the supplement (DHA: 10% of total; ∼500 mg/d; Supplemental Fig. S1). DHA increased RBC EPA levels (*P* < 0.0001), suggesting possible retroconversion of DHA to EPA as observed in plasma in other studies (43, 44), although this change may also reflect a possible contribution of the supplement (EPA: 10% of total; ∼500 mg/d; Supplemental Fig. S1). These data confirm the bioavailability of the supplements.

### Differential prevalence of lipid mediator classes in epidermis, dermis, and plasma

**Figure 1:**
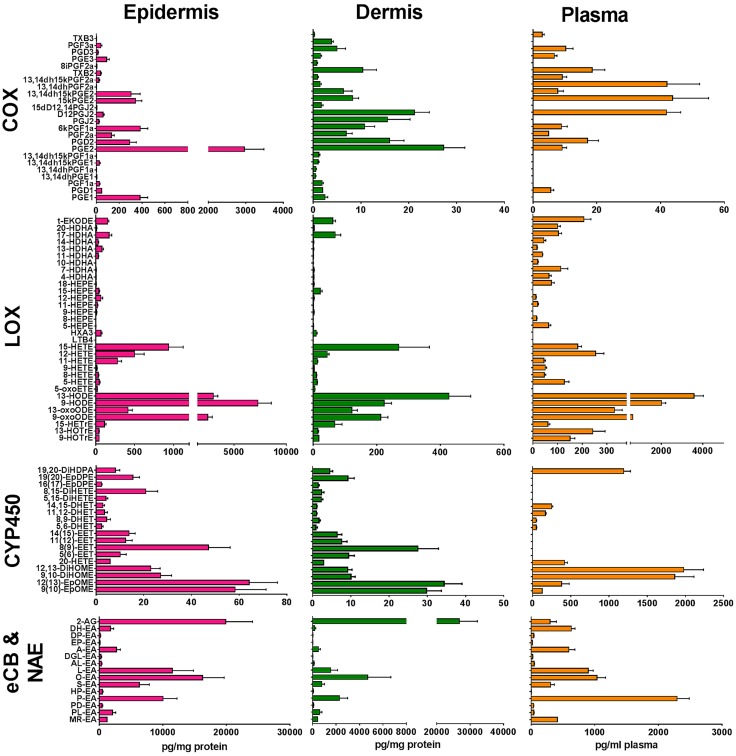
Levels of COX-, LOX-, and CYP450-derived lipid mediators, and eCB and NAE species measured in the epidermis, dermis, and plasma. Baseline levels of lipid mediators were measured in the epidermis, dermis, and plasma of healthy volunteers, without n-3PUFA supplementation or UVR exposure, using UPLC/ESI-MS/MS. Data are shown as means ± sem (*n* = 21). Although grouped as LOX products, some monohydroxy fatty acids can derive from LOX, COX, or CYP450 reactions, or any combination thereof (*e.g.*, 11-HETE, 15-HETE, 13-HODE); trans-epoxyketooctadecenoic acid (t-EKODE) derives from nonenzymatic oxidation. ALEA, alphalinolenoyl ethanolamine; DGLEA, dihomogammalinolenoyl ethanolamine; DHET, dihydroxyeicosatrienoic acid; DiHDPA, dihydroxydocosapentaenoic acid; DiHETE, dihydroxyeicosatetraenoic acid; DiHOME, dihydroxyoctadecenoic acid; DPEA, docosapentaenoyl ethanolamine; EET, epoxyeicosatrienoic acid; EpDPE, epoxydocosapentaenoic acid; EpOME, epoxyoctadecenoic acid; HETrE, hydroxyeicosatrienoic acid; HOTrE, hydroxyoctadecatrienoic acid; HPEA, heptadecanoyl ethanolamine; HX, hepoxilin; LEA, linoleoyl ethanolamine; LT, leukotriene; MEA, myristoyl ethanolamine; OEA, oleoyl ethanolamine; PDEA, pentadecanoyl ethanolamine; PEA, palmitoyl ethanolamine; PLEA, palmitoleoyl ethanolamine; SEA, stearoyl ethanolamine; TX, thromboxane.

The biologic effects of PUFA are partially mediated by their metabolism to eicosanoids, octadecanoids, docosanoids, eCBs, and NAE. We profiled these lipid mediators in skin (epidermis and dermis) and plasma at baseline. We detected and quantitated a total of 89 lipid species at baseline in dermis and epidermis and 66 in plasma (**Fig. 1**). All tissues examined contained similar lipid species, but in different proportions. Notably, the epidermis contained a much higher concentration of COX- and LOX-derived eicosanoids and octadecanoids than dermis. In dermis and epidermis, CYP450-derived lipid mediators were present at a similar concentration to COX-derived mediators, but in plasma, the CYP450-derived lipids were among the most abundant species detected. In both epidermis and dermis, 2-AG was the most abundant eCB species, present at much higher levels than anandamide [arachidonoyl ethanolamine (AEA)]. However, in plasma, 2-AG was a minor species and was less abundant than AEA. Although palmitoyl ethanolamine was the dominant NAE species in plasma, oleoyl ethanolamine was the most abundant NAE in both epidermis and dermis. Interestingly, the sum of EPA metabolites at baseline in unirradiated skin was much lower than for DHA metabolites in all tissues examined, with epidermis showing much higher overall levels than dermis (Supplemental Fig. S3).

### EPA is more efficient in reducing baseline epidermal AA mediators, whereas both EPA and DHA lower NAE

**Figure 2:**
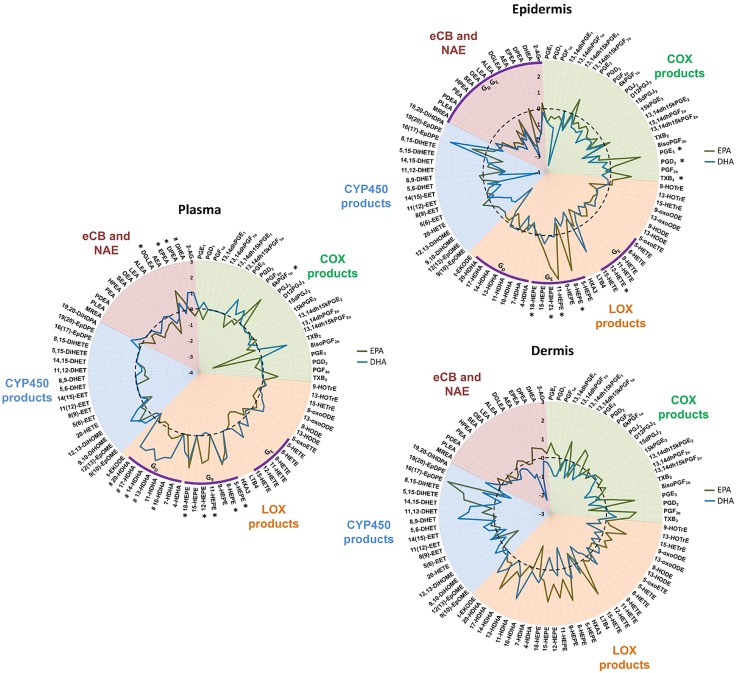
Relative changes in epidermal, dermal, and plasma levels of COX-, LOX-, and CYP450-derived lipid mediators, and eCB and NAE species, following 10-wk supplementation with EPA or DHA. Lipid species were measured after *n*-3PUFA supplementation in healthy human volunteers, without UVR exposure, using UPLC/ESI-MS/MS. Data are shown as log_2_-fold change (black dotted line represents baseline). EPA: *n* = 12 volunteers; DHA: *n* = 9 volunteers. *P < 0.05 post-EPA; ^#^*P* < 0.05 post-DHA. Group changes revealed by iGA with PC <5 × 10^−4^ are represented by purple arcs (*G*_E_ post-EPA, *G*_D_ post-DHA). All data were compared with control unsupplemented tissue. Although grouped as “LOX” products, some monohydroxy fatty acids can derive from LOX, COX, or CYP450 reactions, or any combination thereof (*e.g.*, 11-HETE, 15-HETE, 13-HODE); trans-epoxyketooctadecenoic acid (t-EKODE) derives from nonenzymatic oxidation. ALEA, alphalinolenoyl ethanolamine; DGLEA, dihomogammalinolenoyl ethanolamine; DHET, dihydroxyeicosatrienoic acid; DiHDPA, dihydroxydocosapentaenoic acid; DiHETE, dihydroxyeicosatetraenoic acid; DiHOME, dihydroxyoctadecenoic acid; DPEA, docosapentaenoyl ethanolamine; EET, epoxyeicosatrienoic acid; EpDPE, epoxydocosapentaenoic acid; EpOME, epoxyoctadecenoic acid; HETrE, hydroxyeicosatrienoic acid; HOTrE, hydroxyoctadecatrienoic acid; HPEA, heptadecanoyl ethanolamine; HX, hepoxilin; LEA, linoleoyl ethanolamine; LT, leukotriene; MEA, myristoyl ethanolamine; OEA, oleoyl ethanolamine; PDEA, pentadecanoyl ethanolamine; PEA, palmitoyl ethanolamine; PLEA, palmitoleoyl ethanolamine; SEA, stearoyl ethanolamine; TX, thromboxane.

Global changes in PUFA-derived lipid mediators in epidermis, dermis, and plasma following EPA and DHA supplementation were explored using iGA (37) (Supplemental Table S4). EPA supplementation up-regulated the overall production of EPA-derived hydroxyeicosapentaenoic acid (HEPE) species in both epidermis and plasma (PC = 2 × 10^−7^ and 1.0 × 10^−5^, respectively), and a number of individual HEPE species were significantly increased (*i.e.*, 8-, 11-, 12-, and 18-HEPE, all *P* < 0.025) (**Fig. 2**). EPA was also metabolized by COX, resulting in increased production of epidermal thromboxane B_3_, PGE_3_, and PGD_3_ (all *P* < 0.023). Although the DHA supplement increased concentrations of hydroxydocosahexaenoic acid (HDHA) species in epidermis and plasma (PC = 1.2 × 10^−4^ and 4.2 × 10^−5^, respectively), contrary to EPA, it had no significant effect on the production of any individual epidermal lipid mediator species. It is noteworthy that EPA, but not DHA, significantly reduced the prevalence of AA-derived HETE species in the epidermis (PC = 4.5 × 10^−4^) and to a lesser degree in plasma (PC = 9.5 × 10^−3^). In particular, EPA reduced the epidermal concentration of the chemotactic mediator 12-HETE (*P* = 0.023) (Fig. 2). Neither fatty acid altered the dermal HETE.

Interestingly, both EPA and DHA reduced the overall prevalence of eCB and NAE species in n-3PUFA supplemented epidermis (PC = 3.2 × 10^−5^ and 3.8 × 10^−6^, respectively), but not in either the dermis or plasma. The observed group-wide decreases were not attributed to changes in any individual eCBs and NAE species, whereas dermal mediators showed large variability and neither supplement induced significant changes. Finally, neither fatty acid supplement had any impact upon epidermal CYP450-derived epoxide and diol species (Fig. 2).

In contrast to what was observed in the epidermis, both EPA and DHA increased their respective metabolites in plasma, individually as well as a group change, clearly showing different metabolism compared with skin and offering further evidence for the bioavailability of the supplement. The EPA metabolites 5-, 8-, 11-, 12-, and 18-HEPE, EPEA, and DPEA were all increased (all *P* < 0.017) in plasma (Fig. 2). The DHA-derived 10-, 13-, 14-, 17-, and 20-HDHA, and DHEA (all *P* < 0.031) were increased in plasma following supplementation with DHA (Fig. 2). Neither supplement affected the levels of CYP450-derived epoxy eicosatrienoic acid and dihydroxyeicosatrienoic acid plasma species.

In order to obtain further insight into the differential effects of EPA and DHA, we examined the sum of EPA and DHA metabolites measured in each tissue compartment (epidermis, dermis, and plasma; Supplemental Fig. S3). We observed that the baseline concentrations of EPA-derived lipid species were significantly increased in all 3 tissues post supplementation (*P* = 0.02, *P* = 0.03, *P* = 0.0005 in epidermis, dermis, and plasma, respectively). Conversely, the baseline levels of DHA-derived epidermal and dermal lipid species were less affected by the DHA supplement, and the overall change did not reach statistical significance in skin. However, the DHA supplement elevated the total concentration of DHA-derived mediators found in plasma (*P* = 0.01). Finally, plasma and cutaneous lipid mediators did not show any clear correlations, in either dermis or epidermis, indicating that the nutritional supplements are metabolized and incorporated differently into these tissues; therefore, plasma levels of lipid mediators cannot be used as surrogate markers for cutaneous PUFA metabolism (Supplemental Fig. S4).

As EPA and DHA can exert protective effects through formation of proresolving mediators, the prevalence of RvE1, RvD1, or PDX was also examined. None of these lipids were consistently observed in the skin or plasma samples analyzed, and peaks corresponding to these compounds were below the limit of detection of the assay used. However, a number of peaks corresponding to compounds with the same molecular mass and similar (but not identical) retention times were found at low concentrations in n-3PUFA-supplemented epidermis (Supplemental Fig. S5). This finding could suggest the presence of other polyhydroxy PUFA metabolites, possibly members of the Rv, PD, or maresin families, including positional or geometric isomers or both, whose identity remains to be explored.

### EPA is more effective than DHA in altering production of epidermal lipid mediators after UVR

In order to appreciate the impact of EPA and DHA on the skin’s response to inflammation, we assessed the effect of each supplement on the epidermal and dermal lipid mediators produced 24 and 72 h after a single inflammatory UVR dose of 3× MED (group statistics shown in Supplemental Table S5). The inflammatory UVR challenge caused most significant changes in the epidermal but not dermal mediators (data not shown). These included increased global levels of prostanoids (24 h after UVR) and hydroxy fatty acids (24 and 72 h after UVR), in agreement with our earlier observations in cutaneous blister fluid (29, 45). UVR did not stimulate production of epidermal eCBs, NAE, or CYP450 mediators, and it may be possible that repeated or higher UVR doses, or different inflammatory stimuli, are needed to activate these pathways (18, 33, 46).

**Figure 3:**
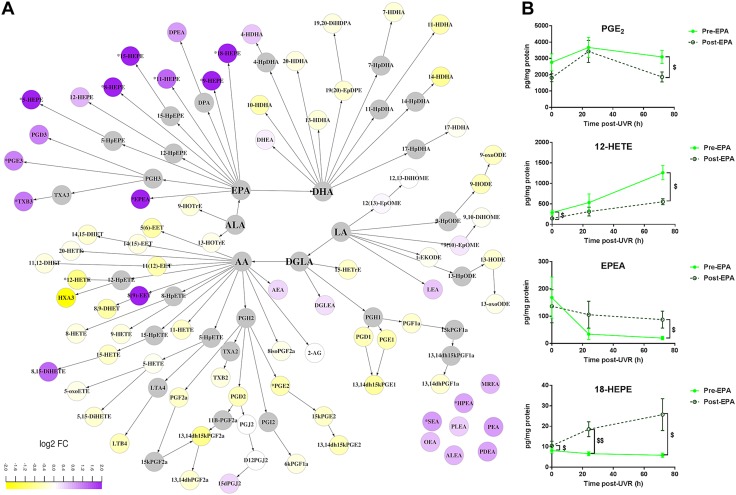
Effect of EPA supplementation on UVR-induced epidermal expression of lipid mediators. Epidermal lipids were measured 72 h after UVR exposure (3× MED), before and after 10-wk supplementation with EPA. *A*) Differences caused by EPA are shown in a metabolic map showing relative changes (log_2_ fold change) from unsupplemented skin. *B*) Selected lipid species affected by EPA (means ± sem, *n* = 12 volunteers). ^$^*P* < 0.05; ^$$^*P* < 0.001 presupplementation *vs.* postsupplementation at each time point. ALEA, alphalinolenoyl ethanolamine; DGLEA, dihomogammalinolenoyl ethanolamine; DHET, dihydroxyeicosatrienoic acid; DiHDPA, dihydroxydocosapentaenoic acid; DiHETE, dihydroxyeicosatetraenoic acid; DiHOME, dihydroxyoctadecenoic acid; DPEA, docosapentaenoyl ethanolamine; EET, epoxyeicosatrienoic acid; EpDPE, epoxydocosapentaenoic acid; EpOME, epoxyoctadecenoic acid; HETrE, hydroxyeicosatrienoic acid; HOTrE, hydroxyoctadecatrienoic acid; HPEA, heptadecanoyl ethanolamine; HX, hepoxilin; LEA, linoleoyl ethanolamine; LT, leukotriene; MEA, myristoyl ethanolamine; OEA, oleoyl ethanolamine; PDEA, pentadecanoyl ethanolamine; PEA, palmitoyl ethanolamine; PLEA, palmitoleoyl ethanolamine; SEA, stearoyl ethanolamine; *t*-EKODE, *trans*-epoxyketooctadecenoic acid; TX, thromboxane.

The EPA supplement did not attenuate the UVR-induced up-regulation of HETE in the epidermis (PC = 0.250 and 0.680, at 24 and 72 h after UVR, respectively; Supplemental Table S5), but it significantly reduced the AA-derived proinflammatory chemotactic 12-HETE (*P* = 0.012) and immunomodulatory PGE_2_ at 72 h after UVR (*P* = 0.028) (**Fig. 3*A*****,** *B*). Furthermore, the EPA-supplemented epidermis showed a group-wise increased production of eCBs and NAE at 72 h after UVR (PC = 8.7 × 10^−6^, compared with unsupplemented skin; Supplemental Table S5) that was also reflected in the up-regulation of individual NAE species such as EPEA, heptadecanoyl ethanolamine, stearoyl ethanolamine, and palmitoyl ethanolamine (all *P* < 0.048) (Fig. 3*A*). Compared to unsupplemented skin, EPA also promoted production of PGE_3_, thromboxane B_3_, and a number of HEPE species (5-, 8-, 9-, 11-, 15-, and 18-HEPE) (all *P* < 0.048) in the epidermis at 24 and 72 h after UVR (Fig. 3). An increase in the linoleic acid–derived 9 (10)-epoxyoctadecenoic acid (*P* = 0.048, 72 h after UVR) and a decrease in the linoleic acid–derived 13-hydroxyoctadecadienoic acid (HODE) (*P* = 0.015, 24 h after UVR) and dihomo γ linolenic acid–derived 9-hydroxyoctadecatrienoic acid (*P* = 0.0017, 24 h after UVR) were also observed (data not shown). Increased dermal production of HEPE was noted at 24 h after UVR (PC = 1.2 × 10^−4^) compared with unsupplemented skin (Supplemental Table S5), although no individual species reached statistical significance.

**Figure 4:**
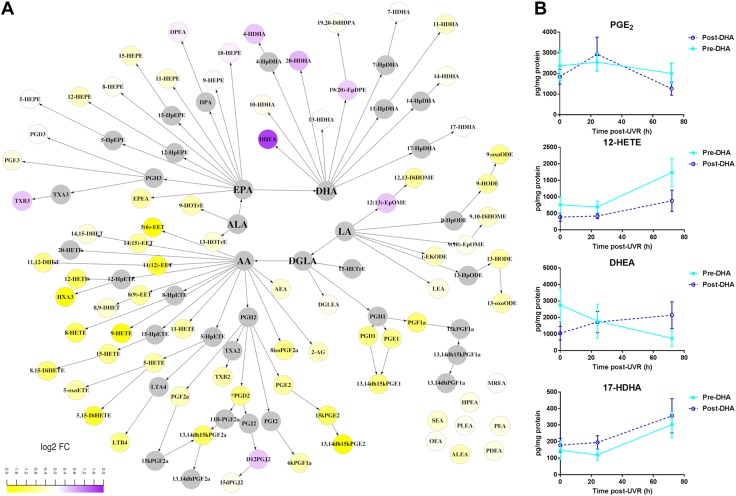
Effect of DHA supplementation on UVR-induced epidermal expression of lipid mediators. Epidermal lipids were measured 72 h after UVR exposure (3× MED), before and after 10 wk of supplementation with DHA. *A*) Differences caused by DHA are shown in a metabolic map showing relative changes (log_2_ fold change) from unsupplemented skin. *B*) Selected species affected by DHA (means ± sem, *n* = 9 volunteers). ALEA, alphalinolenoyl ethanolamine; DGLEA, dihomogammalinolenoyl ethanolamine; DHET, dihydroxyeicosatrienoic acid; DiHDPA, dihydroxydocosapentaenoic acid; DiHETE, dihydroxyeicosatetraenoic acid; DiHOME, dihydroxyoctadecenoic acid; DPEA, docosapentaenoyl ethanolamine; EET, epoxyeicosatrienoic acid; EpDPE, epoxydocosapentaenoic acid; EpOME, epoxyoctadecenoic acid; HETrE, hydroxyeicosatrienoic acid; HOTrE, hydroxyoctadecatrienoic acid; HPEA, heptadecanoyl ethanolamine; HX, hepoxilin; LEA, linoleoyl ethanolamine; LT, leukotriene; MEA, myristoyl ethanolamine; OEA, oleoyl ethanolamine; PDEA, pentadecanoyl ethanolamine; PEA, palmitoyl ethanolamine; PLEA, palmitoleoyl ethanolamine; SEA, stearoyl ethanolamine; *t*-EKODE, *trans*-epoxyketooctadecenoic acid; TX, thromboxane.

Conversely, DHA had a moderate impact on the production of lipid mediators after UVR. Although supplementation with DHA attenuated the UVR-induced global production of HETE (PC = 3.5 × 10^−5^) and increased the production of HDHA (PC = 1.3 × 10^−4^) at 72 h after UVR (Supplemental Table S5), we did not find significant changes in any individual lipid species (**Fig. 4**). However, DHA reduced production of the immunomodulatory PGD_2_ in the epidermis at 72 h after UVR (*P* = 0.008, compared with unsupplemented skin) (Fig. 4*A*). Finally, differently to EPA, DHA did not alter the production of NAE after UVR (PC = 0.089 and 0.001, at 24 and 72 h after UVR, respectively), including its metabolite DHEA.

### EPA and DHA alter expression of lipid metabolizing enzymes in epidermis

**Figure 5:**
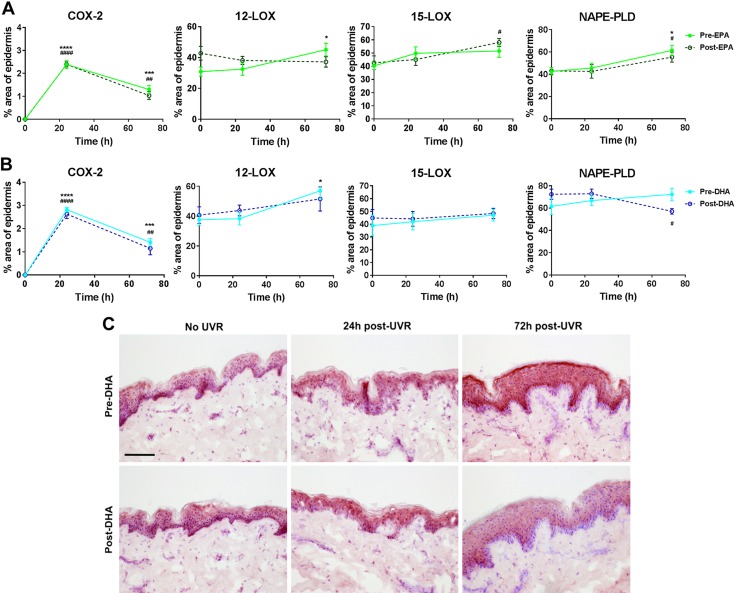
*A*, *B*) UVR-induced changes in lipid metabolizing enzyme expression in the epidermis following 10-wk supplementation with EPA (*A*) or DHA (*B*). Expression of COX-2, 12-LOX, 15-LOX, and NAPE-PLD in unirradiated epidermis and epidermis at 24 and 72 h after UVR exposure (3× MED), both before and after supplementation with EPA or DHA. Expression was assessed by immunohistochemistry, and data are shown as the percentage of epidermis staining positive for enzyme expression (means ± sd; EPA: *n* = 12 and DHA: *n* = 9 volunteers). *P < 0.05; **P < 0.001; ****P < 0.0001 presupplementation *vs.* 0 h presupplementation. ^#^*P* < 0.05; ^##^*P* < 0.01; ^####^*P* < 0.0001 postsupplementation *vs.* 0 h postsupplementation. *C*) Representative photomicrographs of NAPE-PLD expression (red staining in the epidermis) in unirradiated skin and skin 24 and 72 h after UVR exposure (3× MED), both presupplementation and postsupplementation with DHA. Scale bar, 100 µm.

As production of lipid mediators can be regulated by the amount and activity of lipid metabolizing enzymes, as well as availability of precursor fatty acid, we assessed the expression of COX-2, 12-LOX, 15-LOX, and NAPE-PLD proteins by immunohistochemistry in the epidermis at baseline and following UVR, both pre- and post- EPA and DHA supplementation (**Fig. 5**). We focused on assessing changes in the distribution of enzyme staining in the epidermis as this is where we observed most profound lipid mediator changes. Although COX-2 expression was stimulated after UVR (*P* < 0.0001 for all treatments at 24 h), it was not affected by EPA or DHA supplementation. The percentage area of 12-LOX positive epidermis was significantly increased at 72 h after UVR in unsupplemented skin (*P* = 0.03), but not after EPA or DHA supplementation. The percentage area of 15-LOX positive epidermis was not affected by UVR in unsupplemented or DHA-supplemented skin, but EPA supplementation led to a significant increase 72 h after UVR (*P* = 0.01). Interestingly, EPA and DHA demonstrated differential effects on NAPE-PLD expression: whereas UVR increased the % area of epidermis positive for NAPE-PLD in unsupplemented (*P* = 0.04) and EPA-supplemented skin (*P* = 0.04), DHA supplementation resulted in a significantly decreased percentage of epidermal NAPE-PLD expression at 72 h after UVR (*P* = 0.01).

### EPA and DHA supplemented individually do not reduce the erythemal response of human skin to UVR

Because the n-3PUFA supplementation altered the production of lipid mediators after UVR, we measured the effect of EPA and DHA on the skin’s resistance to the inflammatory challenge. Volunteers’ MED and the resolution of UVR-induced erythema over 72 h were assessed before and after n-3PUFA supplementation (Supplemental Fig. S6). Neither EPA nor DHA altered volunteers’ MED or erythema resolution. The EPA data are in agreement with our earlier work reporting no changes in MED following EPA supplementation (4 g/d for 12 wk) (26); there is no previous literature on clinical studies using DHA alone.

### EPA and DHA promote an earlier up-regulation of infiltrating CD4^+^ and CD8^+^ T cells, whereas DHA reduces UVR-induced migration of LCs from the epidermis

**Figure 6:**
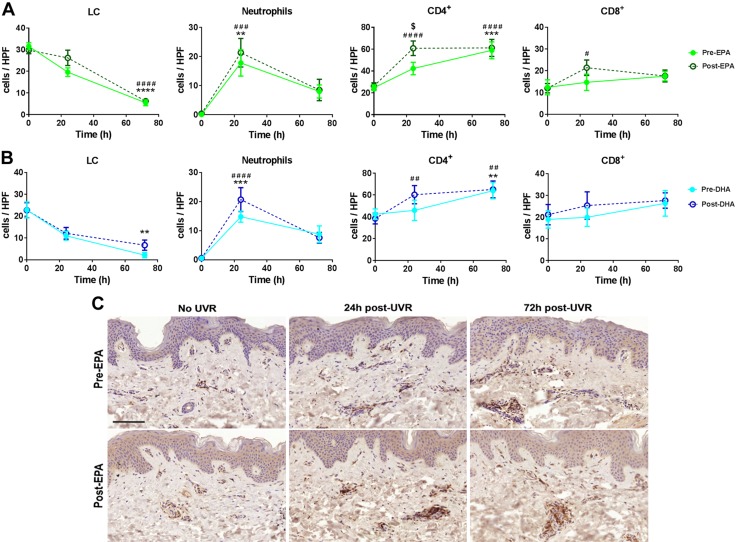
*A*, *B*) UVR-induced changes in immune cell numbers following 10 wk of supplementation with EPA (*A*) or DHA (*B*). The number of LCs in the epidermis and neutrophils and CD4^+^ and CD8^+^ T lymphocytes in the dermis were quantified using immunohistochemistry in unirradiated skin and at 24 and 72 h after UVR exposure (3× MED), both before and after supplementation with EPA or DHA. Data are expressed as number of positively stained cells (means ± sd; EPA *n* = 12 and DHA *n* = 9 volunteers) per HPF. **P < 0.01; ***P < 0.001 presupplementation *vs.* 0 h presupplementation. ^#^*P* < 0.05; ^##^*P* < 0.01; ^###^*P* < 0.001; ^####^*P* < 0.0001 postsupplementation *vs.* 0 h postsupplementation. ^$^*P* < 0.05 postsupplementation *vs.* presupplementation. *C*) Representative photomicrographs of CD4^+^ T cells (brown cells) in unirradiated skin and skin 24 and 72 h after UVR exposure (3× MED), both presupplementation and postsupplementation with EPA. Scale bar, 100 µm.

We evaluated the cellular infiltrate as an indicator of underlying cutaneous inflammation. Infiltration of neutrophils and CD4^+^ and CD8^+^ T lymphocytes were assessed by immunohistochemistry in epidermis and dermis, pre- and postirradiation (24 and 72 h). Dermal neutrophil numbers were significantly increased at 24 h after UVR (*P* = 0.0024, *P* = 0.001; pre-EPA or pre-DHA supplementation, respectively) (**Fig. 6**). Neither n-3PUFA altered this profile (EPA *P* = 0.0004, DHA *P* < 0.0001; 24 h after UVR), although a trend for higher numbers of neutrophils in both EPA- and DHA-supplemented dermis was observed at 24 h after UVR.

Whereas CD4^+^ T-cell numbers in the dermis were increased at 72 h after UVR (*P* < 0.05 for all groups), both EPA- and DHA-supplemented skin showed an earlier peak in CD4^+^ T cells than nonsupplemented skin, reaching statistical significance at 24 h after UVR (EPA *P* < 0.0001, DHA *P* = 0.0082 compared with unirradiated skin). The number of dermal CD8^+^ T cells did not increase significantly 24 or 72 h after UVR in unsupplemented skin (Fig. 6). However, similar to CD4^+^ T cells, EPA-treated dermis showed an earlier peak in CD8^+^ cells compared with untreated dermis. This is seen as a significant increase in CD8^+^ T cells at 24 h after UVR (*P* = 0.01, compared with unirradiated skin) (Fig. 6); a similar trend was observed in DHA-treated skin, but this did not reach statistical significance.

Finally, we assessed the impact of the supplements on the UVR-induced migration of LCs, as their loss from the epidermis contributes to UVR suppression of cell-mediated immunity. The number of LCs was significantly reduced 72 h after UVR in unsupplemented skin (*P* < 0.0001 pre-EPA, *P* = 0.0037 pre-DHA), and following EPA supplementation (*P* < 0.0001; Fig. 6). However, following DHA supplementation, LC migration from the epidermis appeared reduced, which could indicate a delayed response to UVR.

## DISCUSSION

Nutritional supplementation with n-3PUFA has the potential to ameliorate cutaneous inflammation, and in this study, we show that EPA and DHA have differential effects in human epidermis, altering the network of lipid mediators and modulating immune cells in distinct and separate ways. Our findings suggest that EPA and DHA metabolites may be performing different roles in skin, an inference that should be taken into consideration when designing n-3PUFA interventions and treatments for cutaneous disease.

Overall, the concentration of n-3PUFA-derived lipid mediators was higher in epidermis than dermis, and the impact of fatty acid supplementation on lipid mediator biosynthesis was more profound in epidermis, which is indicative of increased n-6 and n-3PUFA metabolism in this outer layer of human skin. Interestingly, the baseline levels of EPA metabolites in epidermis were found to be at least an order of magnitude lower than the DHA-derived lipid species (mean values 319 *vs.* 3136 pg/mg protein). Furthermore, the EPA supplement was far more effective in increasing production of EPA mediators than DHA, the latter having a much less profound effect on its epidermal metabolites (epidermal EPA metabolites increased by 108% following EPA supplementation, whereas epidermal DHA products did not change significantly following DHA supplementation). This feature may be skin specific, as both EPA and DHA supplements were equally effective in significantly increasing their circulating (plasma) metabolites (Supplemental Fig. S4). Currently it is not clear why DHA metabolites would be found in unsupplemented, baseline skin at high concentrations, and their homeostatic role requires further exploration. To reduce the impact of different ethnicities on interindividual variation, this study was limited to white Caucasian volunteers. However, because we have previously found differences in baseline n-3PUFA-derived serum lipids in different skin types (33), it would be interesting in future studies to examine whether EPA and DHA have the same effect in other skin types.

Another clear difference between EPA and DHA was their impact on AA-derived epidermal mediators, with EPA being more efficient in reducing AA products, in particular production of LOX-derived HETE (Fig. 2). This effect of EPA in skin appears to be exerted primarily *via* competition with AA for membrane incorporation and availability for lipid mediator production, as it did not inhibit the baseline expression of COX or LOX protein (Fig. 5). In inflammation, EPA elicited more changes in the lipid mediator network than DHA and attenuated the production of vasodilatory PGE_2_ and chemotactic 12-HETE after UVR, possibly by limiting UVR-induced 12-LOX expression, although it had no impact on inducible COX-2 expression (Fig. 5). Therefore, EPA supplementation may be advantageous to cutaneous conditions characterized by up-regulated LOX products and increased inflammatory infiltrate, such as psoriasis, where long-term EPA intake has been shown to be associated with some clinical improvement (47, 48).

Although DHA had, overall, less impact after UVR, it uniquely reduced the concentration of PGD_2_ (Fig. 4), an immunomodulatory PG that is produced by dendritic cells and plays a central role in cutaneous allergic inflammation (49). This property of DHA may support its potential use to mitigate allergic responses, as shown in an animal model of allergic dermatitis (1). As DHA supplementation appeared to slow the UVR-induced migration of epidermal LC, a source of epidermal PGD_2_, the observed reduction could be caused by altered dendritic cell activity. Changes in PG production are most probably attributed to altered substrate availability, as neither EPA nor DHA inhibited the UVR-induced percentage area of COX-2 protein expression in human epidermis (Fig. 5). Although reports have shown that topically applied DHA inhibited percentage of COX-2 positivity in murine epidermis and that EPA stimulated COX-2 in HaCaT keratinocytes, these effects appear specific to animal skin or immortalized cells (50, 51).

Interestingly, EPA and DHA appeared equally effective in reducing the epidermal global expression of eCB and NAE species (Fig. 2). Although short-term (2 wk) supplementation of mice with DHA has been shown to reduce AEA and increase DHEA in the brain (52), this is the first such study in human skin, suggesting the underlying mechanisms of n-3PUFA-mediated NAE suppression in epidermis warrants further investigation. The distribution of epidermal NAPE-PLD protein expression was not altered by EPA or DHA in unirradiated skin (Fig. 5), suggesting either the potential involvement of other NAE biosynthetic reactions or reduced glycerophospholipid substrate availability (53). However, EPA, but not DHA, induced the overall epidermal production of NAE in UVR-challenged skin (Figs. 3 and 4). This finding can, at least in part, be attributed to the lower percentage area of epidermis expressing NAPE-PLD found in DHA-supplemented skin after UVR, an effect that could result in reduced production of NAE (Fig. 5). A NAPE-PLD knockout animal model has shown widespread effects on brain lipids, including inhibition of NAE and an increase of PGE_2_ (54). Therefore, inhibition of NAPE-PLD by DHA or its metabolites may have broader implications for the cutaneous lipidome, not necessarily restricted to reduced formation of NAE. Conditions such as irritant dermatitis, where eCBs and NAE appear up-regulated, may benefit from DHA interventions (6, 18, 55).

The EPA- and DHA-induced changes in the cutaneous lipid mediator network and consequent impact on chemotactic lipid species may also explain the observed increase in recruitment of CD4^+^ and CD8^+^ T cells at 24 h after UVR (Fig. 6). n-3PUFA modification of cellular lipids can modulate the biophysical properties and composition of membrane microdomains and alter the associated signaling pathways, whereas PUFA-derived lipid mediators acting through cell surface receptors affect T-lymphocyte activation, differentiation, proliferation, motility, and homing events [reviewed in (11)]. Furthermore, EPA and DHA have the potential to alter expression of cutaneous adhesion molecules (*e.g.*, intercellular adhesion molecule 1 (ICAM-1), VCAM-1, E-selectin) important for recruitment of T cells, although this has not yet been shown in human skin (56, 57). Nonetheless, this increase in T-cell infiltration at 24 h after UVR is an unanticipated and intriguing finding of supplementation by n-3PUFA, which display predominantly anti-inflammatory activities.

The implications of an augmented T-cell infiltrate during UVR-induced inflammation in EPA- and DHA-supplemented skin are uncertain: CD4^+^ T cells can support the development of CD8^+^ T cells, typically regarded as cytotoxic; so, the EPA-induced promotion of an earlier CD8^+^ T-cell infiltrate may condition human skin to become more resistant to inflammation and infections (58, 59). In support of a protective role of n-3PUFA, it has been shown that DHA-induced suppression of allergic reactions was accompanied by increased forkhead box P3 (*FoxP3*)^+^CD4^+^ regulatory T cells, and intradermal injection of regulatory T cells could inhibit skin inflammation (1, 60). Potentially, earlier recruitment of CD4^+^ and CD8^+^ lymphocytes might be followed by earlier resolution of these infiltrates; further time course studies beyond 72 h could be revealing.

Collectively, our findings show that EPA and DHA are differentially metabolized in human skin, both under basal conditions and in inflammation, leading to separate changes in the cutaneous mediator lipidome, which impacts the local microenvironment. These differential activities could be harnessed to appropriately target varying types of cutaneous inflammation; however, further work is needed to assess the exact activity of relevant lipid mediators.

## Supplementary Material

This article includes supplemental data. Please visit *http://www.fasebj.org* to obtain this information.

## Abbreviations

ω-3: n-3
AA: arachidonic acid
AEA: arachidonoyl ethanolamine
AG: arachidonoyl glycerol
COX: cyclooxygenase
CYP450: cytochrome P450
DHA: docosahexaenoic acid
DHEA: docosahexaenoyl ethanolamine
eCB: endocannabinoid
EPA: eicosapentaenoic acid
EPEA: eicosapentaenoyl ethanolamine
FAME: fatty acid methyl ester
GC: gas chromatography
HDHA: hydroxydocosahexaenoic acid
HEPE: hydroxyeicosapentaenoic acid
HETE: hydroxyeicosatetraenoic acid
HODE: hydroxyoctadecadienoic acid
HPF: high-power field
iGA: iterative group analysis
LC: Langerhans cell
LOX: lipoxygenase
MED: minimum erythemal dose
NAE: *N*-acyl ethanolamine
NAPE-PLD: *N*-acyl phosphatidylethanolamine–specific phospholipase D
PC: enrichment score
PD: protectin
PG: prostaglandin
PUFA: polyunsaturated fatty acid
RBC: red blood cell
Rv: resolvin
UPLC/ESI-MS/MS: ultraperformance liquid chromatography with electrospray ionization and tandem mass spectrometry
UVR: UV radiation
