# Supplementary material for: Dynamics of the human skin mediator lipidome in response to dietary ω-3 fatty acid supplementation
Source: FASEB J. 2019 Oct 29;33(11):13014–27. doi: 10.1096/fj.201901501R (PMC6902719; doi:10.1096/fj.201901501R)
Supplement: Supplementary file 1 [file fj.201901501R.sf1.docx]

**SUPPLEMENTARY FIGURES**

**
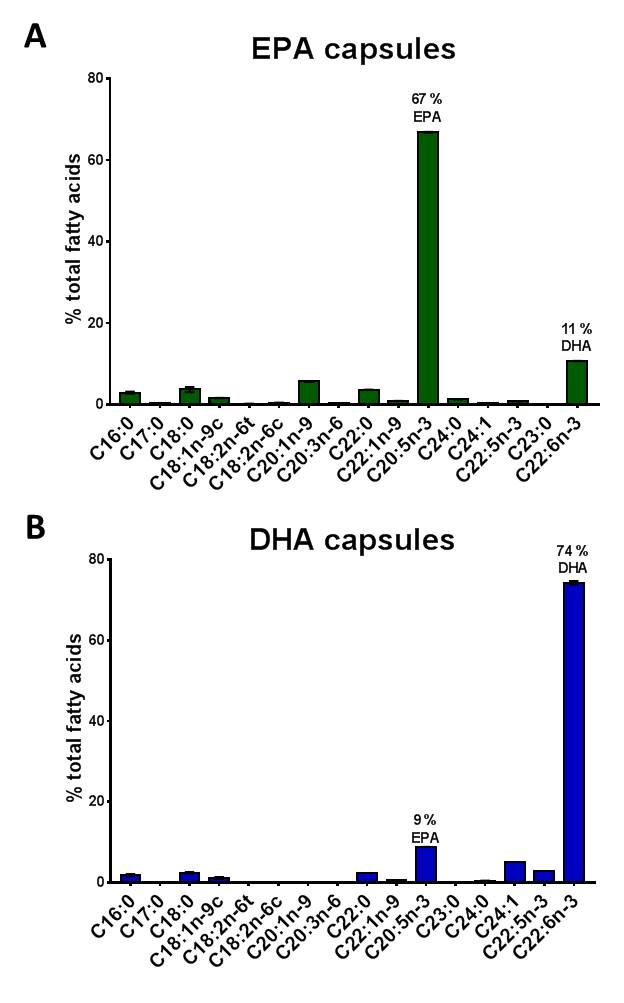
**

**Supplementary Figure S1. Fatty acid analysis of EPA (A) and DHA (B) supplement capsules.** Capsule contents were analysed by gas chromatography (GC-FID) to confirm fatty acid profiles. Individual fatty acid contents are expressed as percentage of total fatty acids (data shown as mean ± SD, n=3 independent analyses).

**
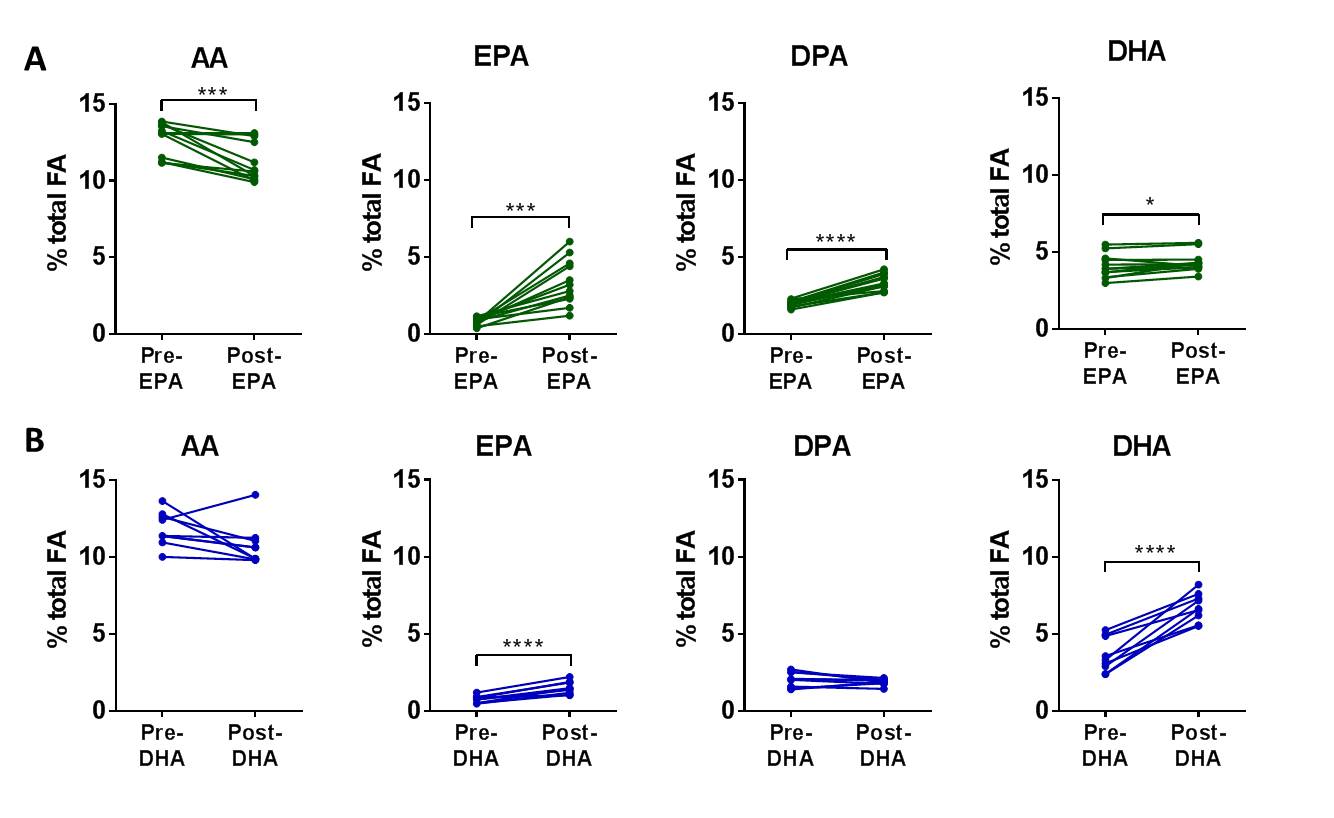
**

**Supplementary Figure S2. Red blood cell fatty acid analysis following 10 weeks’ supplementation with EPA (n=12) (A) or DHA (n=9) (B).** The fatty acid profile of volunteers’ red blood cells was analysed by gas chromatography (GC-FID) to confirm study compliance and supplement bioavailability. Arachidonic acid (AA), eicosapentaenoic acid (EPA), docosahexaenoic acid (DHA) and docosapentaenoic acid (DPA) profiles are expressed as percentage of total fatty acids per individual; *p<0.05, ***p<0.001, ****p<0.0001 comparing pre- vs post-supplementation.


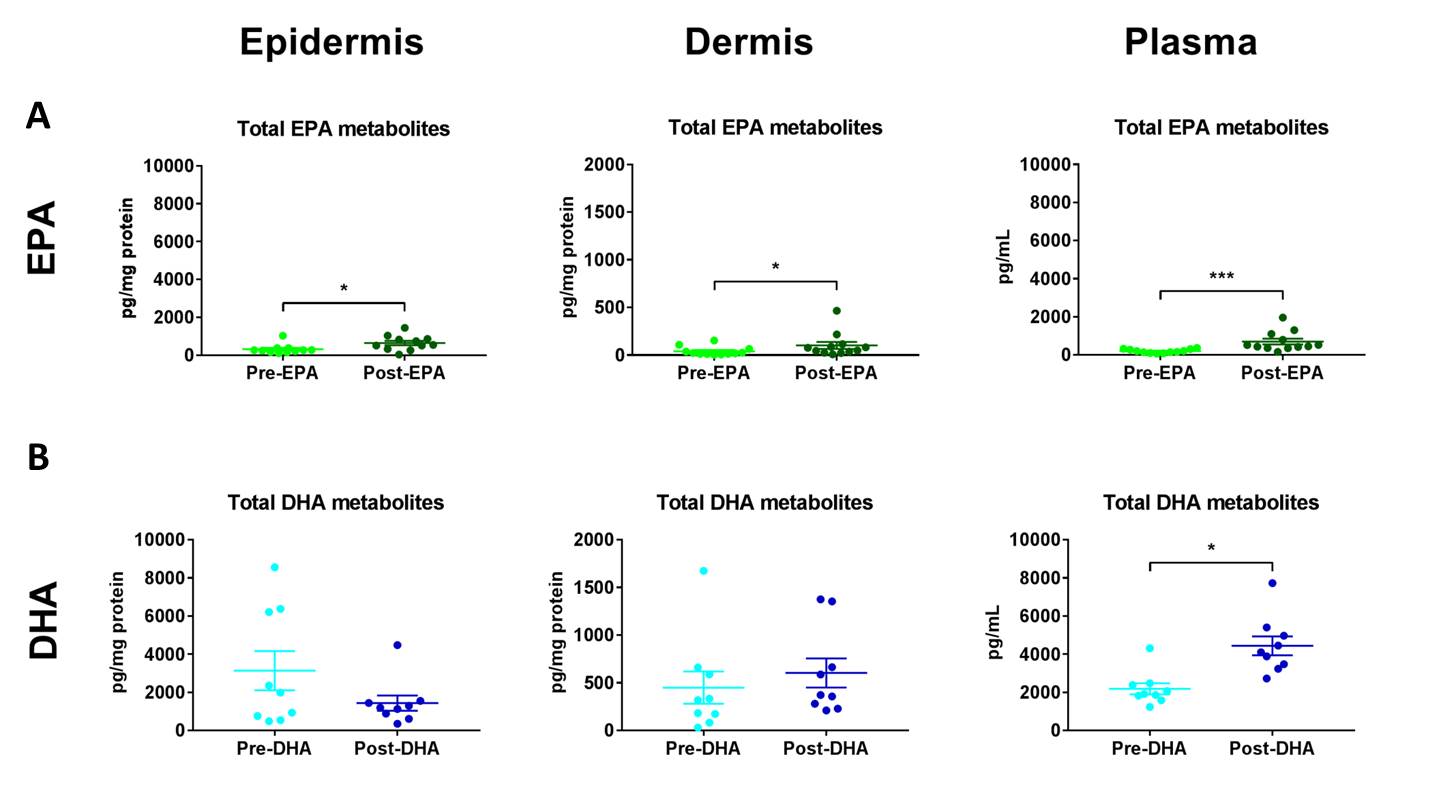


**Supplementary Figure S3. The sum of EPA- and DHA-derived mediators in epidermis, dermis and plasma before and after supplementation.** Lipid metabolites measured by UPLC-MS/MS in epidermis, dermis (pg/mg protein) and plasma (p**g/mL), were summed pre- and post- 10 weeks’ supplementation with EPA (A) or DHA (B**), respectively. *p<0.05, ***p<0.001 Wilcoxon matched-pairs signed rank test, data shown as individual points with bars representing mean±SEM (EPA study n=12, DHA study n=9).


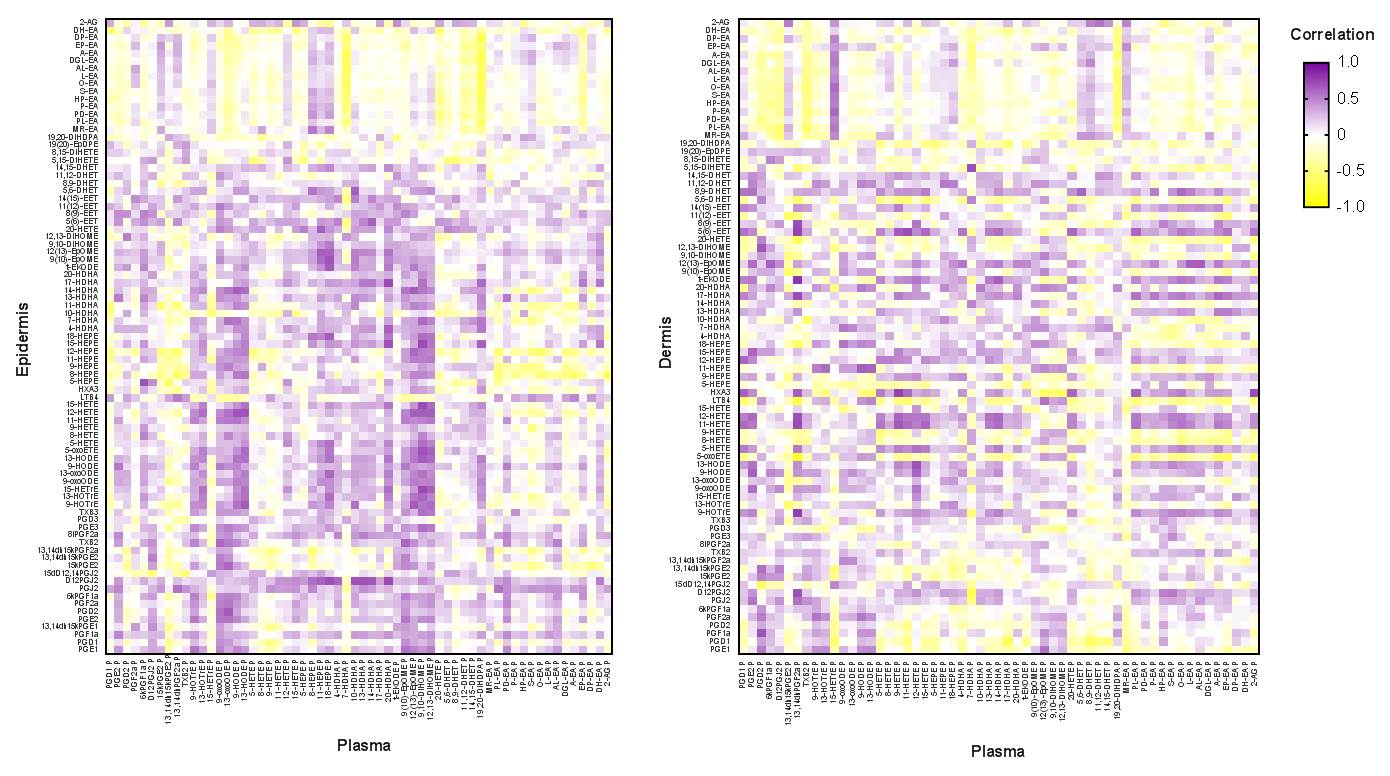


**Supplementary Figure S4. Correlations between baseline levels of lipid mediators measured in plasma, epidermis and dermis.** Lipids in plasma, epidermis and dermis were measured at baseline (pre-supplementation, without UVR challenge) using UPLC/ESI-MS/MS and correlation analysis performed. Data are shown as Spearman correlation coefficient (n=21).


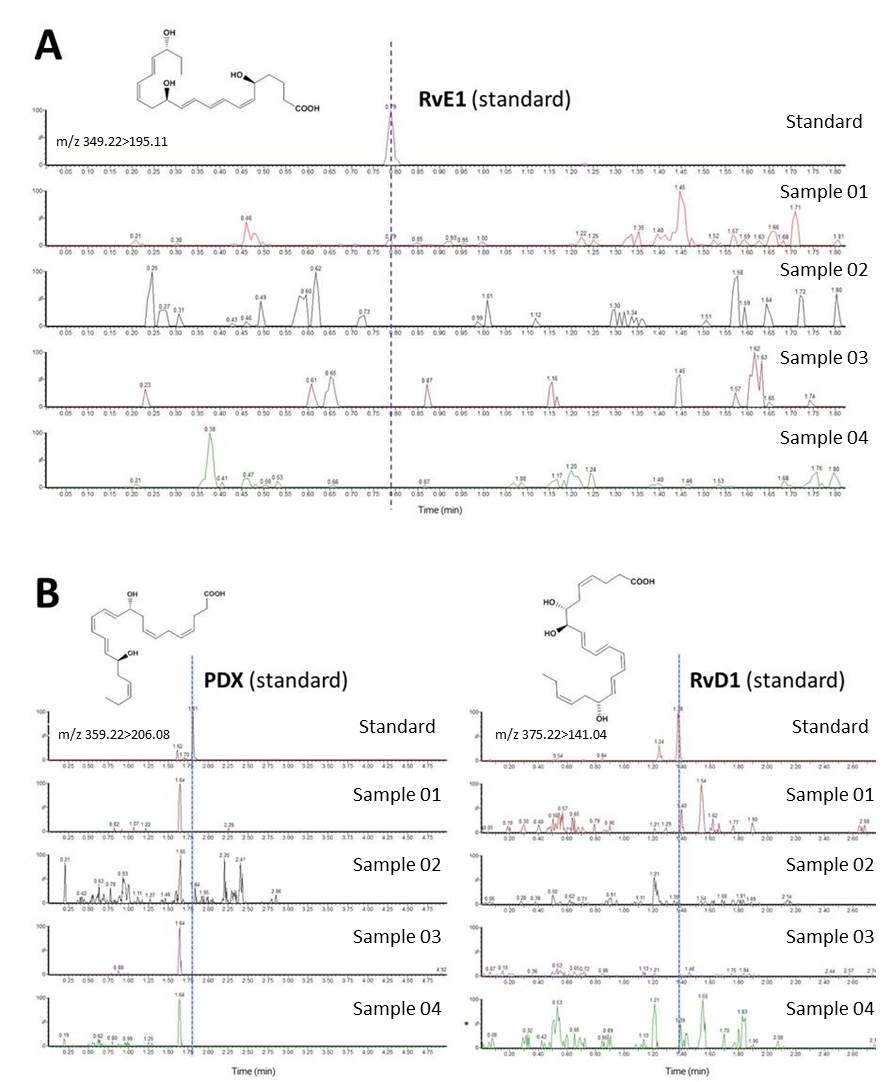


**Supplementary Figure S5. Representative chromatograms of resolvin and protectin species detected in EPA (A) and DHA (B) supplemented epidermis.** Resolvins and protectins were identifiable in standards (RvE1, RvD1 and PDX) but not epidermis samples following 10 weeks’ supplementation with EPA or DHA. The presence of peaks with the same molecular mass and similar (but not identical) retention times suggests the presence of positional and/or geometric isomers, whose identity remains to be explored.

**
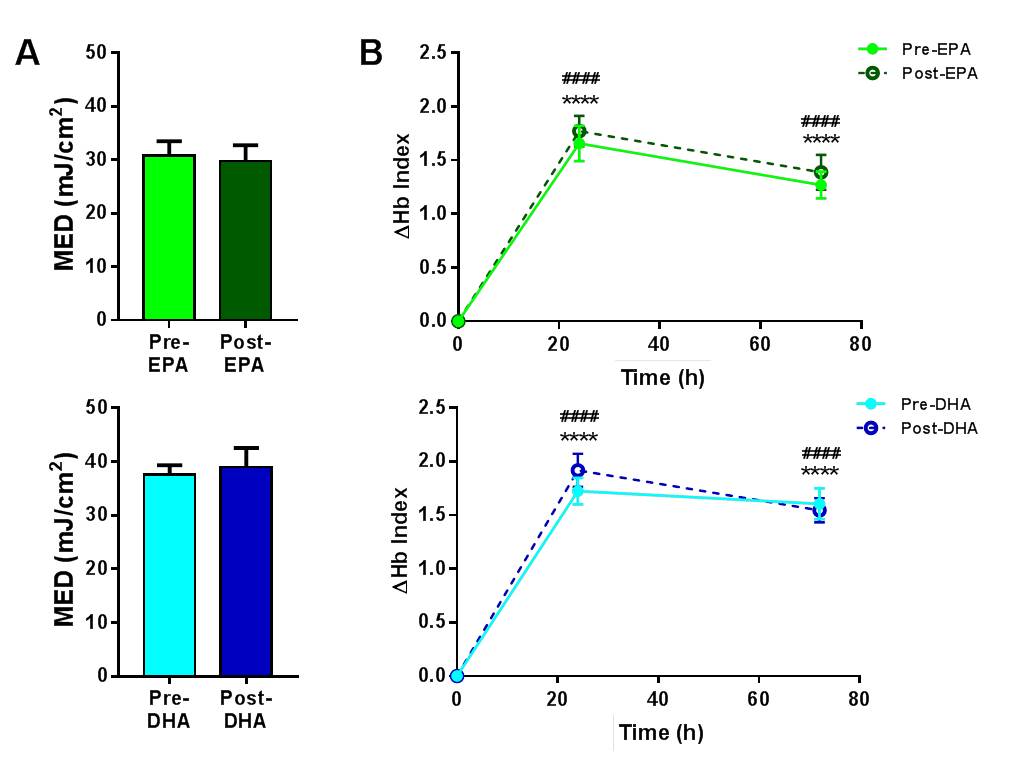
**

**Supplementary Figure S6. UVR-erythema response following 10 weeks’ of supplementation with EPA or DHA**. The minimum dose of UVR required to induce erythema (minimal erythemal dose; MED) was measured in each volunteer before and after 10 weeks supplementation with EPA or DHA (**A**). Also, each individual was given a UVR challenge of 3xMED and the haemoglobin index (∆Hb Index) was monitored over 72 h (**B**). Data are expressed as mean ± SEM; EPA n=12 and DHA n=9 volunteers; ****p<0.0001 pre-supplementation vs 0 h pre-supplementation, ^####^p<0.0001 post-supplementation vs 0 h post-supplementation.
