# Supplementary material for: Dynamics of the human skin mediator lipidome in response to dietary ω-3 fatty acid supplementation
Source: FASEB J. 2019 Oct 29;33(11):13014–27. doi: 10.1096/fj.201901501R (PMC6902719; doi:10.1096/fj.201901501R)
Supplement: Supplementary file 2 [file fj.201901501R.st1.docx]

**SUPPLEMENTARY TABLES**

**Supplementary Table S1:** Mass spectrometry conditions for UPLC/ESI-MS/MS analysis of eicosanoids and related species.

| **Compound** | **MRM** | **Cone**  **voltage (V)** | **Collision energy (eV)** |
| --- | --- | --- | --- |
| PGD_1_ | 353.22>317.23 | 12 | 12 |
| PGE_1_ | 353.22>317.23 | 12 | 12 |
| 6-keto PGF_1α_ | 369.16>163.09 | 12 | 24 |
| 13,14-dihydro-15-keto PGF_1α_ | 355.22>193.13 | 20 | 30 |
| PGF_1α_ | 355.22>311.23 | 14 | 24 |
| 13,14-dihydro PGE_1_ | 355.22>337.24 | 18 | 16 |
| 13,14-dihydro-15-keto PGE_1_ | 353.22>335.24 | 12 | 14 |
| PGB_2_-*d*4 (standard) | 337.22>179.09 | 12 | 20 |
| PGD_2_ | 351.22>271.20 | 24 | 16 |
| PGE_2_ | 351.22>271.20 | 24 | 16 |
| 15-keto PGE_2_ | 349.16>113.07 | 14 | 20 |
| 13,14-dihydro PGF_2α_ | 355.22>311.23 | 14 | 24 |
| 13,14-dihydro-15-keto PGF_2α_ | 353.22>113.06 | 10 | 26 |
| PGF_2α_ | 353.22>193.08 | 12 | 24 |
| 8-iso PGF_2α_ | 353.22>193.08 | 12 | 24 |
| PGJ_2_ | 333.16>271.21 | 14 | 16 |
| Δ^12^-PGJ_2_ | 333.16>271.21 | 14 | 16 |
| 15-deoxy-Δ^12,14^-PGJ_2_ | 315.16>271.15 | 12 | 14 |
| TXB_2_ | 369.22>169.01 | 18 | 18 |
| 13,14-dihydro PGF_1α_ | 357.22>113.06 | 4 | 32 |
| 13,14-dihydro-15-keto PGE_2_ | 351.22>333.24 | 12 | 12 |
| TXB_3_ | 367.16>169.02 | 16 | 14 |
| PGD_3_ | 349.16>269.19 | 10 | 16 |
| PGE_3_ | 349.16>269.19 | 10 | 16 |
| PGF_3α_ | 351.22>193.09 | 2 | 22 |
| 9-HODE | 295.22>171.09 | 16 | 16 |
| 13-HODE | 295.22>195.15 | 2 | 18 |
| 15-HETrE | 321.29>303.23 | 2 | 14 |
| 5-HETE | 319.22>115.02 | 14 | 14 |
| 8-HETE | 319.22>155.03 | 10 | 14 |
| 9-HETE | 319.22>123.09 | 16 | 14 |
| 11-HETE | 319.22>167.06 | 14 | 14 |
| 12-HETE | 319.22>179.09 | 20 | 14 |
| 12-HETE-*d*8 (internal standard) | 327.27>184.33 | 20 | 16 |
| 15-HETE | 319.22>175.13 | 4 | 14 |
| 20 HETE | 319.22>245.20 | 4 | 14 |
| 5(6)-EET | 319.22>191.20 | 4 | 10 |
| 8(9)-EET | 319.22>155.03 | 10 | 14 |
| 11(12)-EET | 319.22>167.06 | 14 | 14 |
| 14(15)-EET | 319.22>175.13 | 4 | 14 |
| 5-HEPE | 317.15>115.02 | 16 | 12 |
| 8-HEPE | 317.22>155.03 | 26 | 12 |
| 9-HEPE | 317.22>149.11 | 20 | 14 |
| 11-HEPE | 317.22>167.07 | 12 | 12 |
| 12-HEPE | 317.16>179.10 | 28 | 12 |
| 15-HEPE | 317.22>175.13 | 8 | 14 |
| 18-HEPE | 317.22>215.20 | 12 | 14 |
| 5,6-DHET | 337.22>145.01 | 8 | 16 |
| 8,9-DHET | 337.22>127.08 | 8 | 16 |
| 11,12-DHET | 337.29>167.06 | 2 | 18 |
| 14,15-DHET | 337.22>207.10 | 18 | 16 |
| 5-oxo-ETE | 317.22>203.23 | 14 | 18 |
| LTB_4_ | 335.22>195.10 | 12 | 14 |
| RvE_1_ | 349.22>195.11 | 14 | 16 |
| RvD_1_ | 375.22>141.04 | 18 | 12 |
| RvD_2_ | 375.22>175.07 | 2 | 22 |
| 4-HDHA | 343.22>101.96 | 8 | 12 |
| 7-HDHA | 343.22>141.04 | 6 | 14 |
| 10-HDHA | 343.29>153.08 | 2 | 16 |
| 11-HDHA | 343.29>193.87 | 2 | 12 |
| 13-HDHA | 343.29>193.15 | 2 | 12 |
| 14-HDHA | 343.22>161.08 | 12 | 14 |
| 17-HDHA | 343.22>201.15 | 14 | 14 |
| 20-HDHA | 343.22>241.21 | 2 | 12 |
| PDX | 359.22>206.08 | 18 | 16 |
| MaR_1_ | 359.22>177.08 | 16 | 16 |
| 9 OxoODE | 293.22>185.15 | 14 | 18 |
| 13 OxoODE | 293.26>113.13 | 16 | 20 |
| 9 HOTrE | 293.24>171.10 | 20 | 16 |
| 13 HOTrE | 293.24>195.30 | 12 | 16 |
| 9(10) EpOME | 295.22>171.09 | 16 | 16 |
| 12(13)EpOME | 295.22>195.15 | 2 | 18 |
| Trans EKODE | 309.22>209.15 | 16 | 10 |
| 9,10 DiHOME | 313.29>201.13 | 16 | 20 |
| 12,13 DiHOME | 313.28>183.98 | 16 | 20 |
| 8(9) EET-*d*11 (internal standard) | 330.34>155.09 | 14 | 12 |
| 8,9 DHET-*d*11 (internal standard) | 348.38>127.06 | 16 | 24 |
| HXA3 | 335.28>273.27 | 16 | 12 |
| 5,15 DiHETE | 335.29>115.09 | 12 | 12 |
| 8,15 DiHETE | 335.29>155.02 | 22 | 16 |
| 16(17) EpDPE | 343.29>233.22 | 14 | 12 |
| 19(20) EpDPE | 343.29>285.24 | 18 | 12 |
| 19,20 DiHDPA | 361.28>273.26 | 18 | 16 |

**Supplementary Table S2:** Mass spectrometry conditions for UPLC/ESI-MS/MS analysis of endocannabinoids and *N*-acyl ethanolamines.

| **Compound** | **MRM** | **Cone voltage (V)** | **Collision energy (eV)** |
| --- | --- | --- | --- |
| AEA-*d*8 (standard) | 356.29>63.10 | 22 | 14 |
| 2-AG-*d*8 (standard) | 387.34>293.20 | 43 | 10 |
| MEA | 272.29>62.06 | 28 | 12 |
| PDEA | 286.35>62.06 | 28 | 12 |
| POEA | 298.35>62.06 | 26 | 14 |
| PEA | 300.29>62.06 | 58 | 12 |
| HEA | 314.35>62.09 | 40 | 12 |
| STEA | 328.29>62.06 | 74 | 14 |
| OEA | 326.29>62.06 | 76 | 14 |
| LEA | 324.29>62.06 | 66 | 14 |
| ALEA | 322.29>62.09 | 62 | 14 |
| DGLEA | 350.35>62.09 | 32 | 14 |
| AEA | 348.29>62.06 | 66 | 16 |
| EPEA | 346.29>62.06 | 62 | 16 |
| DPEA | 374.35>62.06 | 32 | 16 |
| DHEA | 372.29>62.06 | 12 | 16 |
| 2-AG | 379.29>287.25 | 12 | 14 |

**Supplementary Table S3:** Metabolite groupings used for the identification of global changes using iterative group analysis (iGA) and double boundary iterative group analysis (db-iGA).

**Group 1 (code COX):** PGD_1_, PGF_1α_, 13,14dhPGE_1_, 13,14dhPGF_1α_, 13,14dh15kPGE_1_, 13,14dh15kPGF_1α_, PGE_2_, PGD_2_, PGF_2α_, 6kPGF_1α_, PGJ_2_, D12PGJ_2_, 15dD12,14PGJ_2_, 15kPGE_2_, 13,14dh15kPGE_2_, 13,14dhPGF_2α_, 13,14dh15kPGF_2α_, TXB_2_, 8iPGF_2α_, PGE_3_, PGD_3_, PGF_3α_, TXB_3_.

**Group 2 (code LOX):** 9-HOTrE,13-HOTrE, 15-HETrE, 9-oxoODE, 13-oxoODE, 9-HODE, 13-HODE, 5-oxoETE, 5-HETE, 8-HETE, 9-HETE, 11-HETE, 12-HETE, 15-HETE, LTB4, HXA3, 5-HEPE, 8-HEPE, 9-HEPE, 11-HEPE, 12-HEPE, 15-HEPE, 18-HEPE, 4-HDHA, 7-HDHA, 10-HDHA, 11-HDHA, 13-HDHA, 14-HDHA, 17-HDHA, 20-HDHA, t-EKODE.

**Group 3 (code CYP):** 9(10)-EpOME, 12(13)-EpOME, 9,10-DiHOME, 12,13-DiHOME, 20-HETE, 5(6)-EET, 8(9)-EET, 11(12)-EET, 14(15)-EET, 5,6-DHET, 8,9-DHET, 11,12-DHET, 14,15-DHET, 5,15-DiHETE, 8,15-DiHETE, 16(17)-EpDPE, 19(20)-EpDPE, 19,20-DiHDPA.

**Group 4 (code ENDO+NAE):** MR-EA, PL-EA, PD-EA, P-EA, HP-EA, S-EA, O-EA, L-EA, AL-EA, DGL-EA, A-EA, EP-EA, DP-EA, DH-EA, 2-AG.

**Group 5 (code (NAE):** MR-EA, PL-EA, PD-EA, P-EA, HP-EA, S-EA, O-EA, L-EA, AL-EA, DGL-EA, A-EA, EP-EA, DP-EA, DH-EA.

**Group 6 (code HDHA):** 4-HDHA, 7-HDHA, 10-HDHA, 11-HDHA, 13-HDHA, 14-HDHA, 17-HDHA, 20-HDHA.

**Group 7 (code HEPE):** 5-HEPE, 8-HEPE, 9-HEPE, 11-HEPE, 12-HEPE, 15-HEPE, 18-HEPE.

**Group 8 (code HETE):** 5-HETE, 8-HETE, 9-HETE, 11-HETE, 12-HETE, 15-HETE.

**Supplementary Table S4:** Global changes in lipid mediators measured in epidermis, dermis and plasma, before and after a 10-week supplementation with (**A**) EPA and (**B**) DHA. Global changes were identified by iterative group analysis (iGA). The enrichment score (PC) reflects the global changes in the concentration of lipids in each group; a PC value of <5x10^-4^ was considered significant (significant changes marked in red or blue). Note each group was tested both for upregulation and downregulation; in principle, it is possible for a group to be significantly changed in both directions in the same experiment when subsets of the group show opposite behaviour.

| **A. EPA** | **PC – upregulated** after EPA supplementation | | |
| --- | --- | --- | --- |
| **GROUP** | **EPIDERMIS** | **DERMIS** | **PLASMA** |
| G1—COX | 0.058 | 0.30 | 0.14 |
| G2—LOX | 6.4x10^-3^ | 6.5x10^-3^ | 8.7x10^-3^ |
| G3—CYP | 6.5x10^-3^ | 0.32 | 0.39 |
| G4—ENDO+NAE | 0.34 | 0.037 | 0.22 |
| G5—NAE | 0.37 | 0.052 | 0.20 |
| G6—HDHA | 0.018 | 0.27 | 0.065 |
| G7—HEPE | **2.0x10^-7^** | 5.7x10^-3^ | **1.0x10^-5^** |
| G8—HETE | 0.84 | 0.10 | 0.86 |
|  | **PC – downregulated** after EPA supplementation | | |
| **GROUP** | **EPIDERMIS** | **DERMIS** | **PLASMA** |
| 1—COX | 0.094 | 0.12 | 0.12 |
| 2—LOX | 0.053 | 0.051 | 0.32 |
| 3—CYP | 0.070 | 0.10 | 0.031 |
| 4—ENDO+NAE | **3.2x10^-5^** | 0.024 | 0.057 |
| 5—NAE | **7.9x10^-5^** | 0.019 | 0.033 |
| 6—HDHA | 0.42 | 0.17 | 0.21 |
| 7—HEPE | 0.83 | 0.85 | 0.84 |
| 8—HETE | **4.5x10^-4^** | 0.089 | 9.5x10^-3^ |

| **B. DHA** | **PC – upregulated** after DHA supplementation | | |
| --- | --- | --- | --- |
| **GROUP** | **EPIDERMIS** | **DERMIS** | **PLASMA** |
| 1—COX | 0.083 | 0.74 | 0.073 |
| 2—LOX | **9.0x10^-7^** | 0.077 | 4.4x10^-3^ |
| 3—CYP | 0.20 | 6.7x10^-3^ | 0.16 |
| 4—ENDO+NAE | 0.99 | 0.026 | 0.64 |
| 5—NAE | 1.00 | 0.036 | 0.68 |
| 6—HDHA | **1.2x10^-4^** | 4.6x10^-3^ | **4.2x10^-5^** |
| 7—HEPE | 3.6x10^-3^ | 0.44 | 0.020 |
| 8—HETE | 0.059 | 0.25 | 0.38 |
|  | **PC – downregulated** after DHA supplementation | | |
| **GROUP** | **EPIDERMIS** | **DERMIS** | **PLASMA** |
| 1—COX | 1.9x10^-3^ | 4.2x10^-3^ | 0.12 |
| 2—LOX | 1.00 | 0.48 | 0.73 |
| 3—CYP | 0.077 | 0.16 | 0.023 |
| 4—ENDO+NAE | **3.8x10^-6^** | 0.033 | 0.025 |
| 5—NAE | **1.2x10^-6^** | 0.013 | 0.014 |
| 6—HDHA | 0.90 | 0.67 | 0.98 |
| 7—HEPE | 0.84 | 0.22 | 0.78 |
| 8—HETE | 0.38 | 0.17 | 0.034 |

**Supplementary Table S5:** EPA (**A**) and DHA (**B**) induced global changes in epidermal and dermal lipid mediators, at 24 h and 72 h after an inflammatory UVR treatment, compared to un-supplemented skin at each time point (10-week supplementation with EPA or DHA). Global changes were identified by iterative group analysis (iGA). The enrichment score (PC) reflects the global changes in the concentration of lipids in each group; a value of <5x10^-4^ was considered significant (significant changes marked in red or blue). Note each group was tested both for upregulation and downregulation; in principle, it is possible for a group to be significantly changed in both directions in the same experiment when subsets of the group show opposite behaviour.

| **A. EPA** | **PC – upregulated** | | | |
| --- | --- | --- | --- | --- |
|  | **EPIDERMIS** | | **DERMIS** | |
| **GROUP** | **24h post UVR** | **72h post UVR** | **24h post UVR** | **72h post UVR** |
| 1—COX | 0.089 | 0.26 | 0.013 | 0.072 |
| 2—LOX | 0.016 | 0.068 | 0.15 | 0.48 |
| 3—CYP | 0.33 | 0.029 | 0.63 | 0.085 |
| 4—ENDO+NAE | 0.019 | **8.7x10^-6^** | 0.19 | 0.012 |
| 5—NAE | 0.026 | **2.4x10^-5^** | 0.091 | 2.1x10^-3^ |
| 6—HDHA | 0.34 | 0.53 | 0.28 | 0.87 |
| 7—HEPE | **2.0x10^-7^** | **1.4x10^-6^** | **1.3x10^-4^** | 1.1x10^-3^ |
| 8—HETE | 0.25 | 0.68 | 0.49 | 0.53 |
|  | **PC – downregulated** | | | |
|  | **EPIDERMIS** | | **DERMIS** | |
| **GROUP** | **24h post UVR** | **72h post UVR** | **24h post UVR** | **72h post UVR** |
| 1—COX | 0.13 | 2.4x10^-3^ | 0.24 | 0.077 |
| 2—LOX | 0.074 | 0.067 | 0.061 | 1.9x10^-3^ |
| 3—CYP | 0.027 | 0.10 | 0.051 | 0.11 |
| 4—ENDO+NAE | 0.030 | 0.42 | 0.045 | 0.49 |
| 5—NAE | 0.044 | 0.45 | 0.064 | 0.53 |
| 6—HDHA | 0.13 | 0.038 | 0.44 | 0.028 |
| 7—HEPE | 0.98 | 1.00 | 0.92 | 0.92 |
| 8—HETE | 0.18 | 0.052 | 0.014 | 7.1x10-13 |

| **B. DHA** | **PC – upregulated** | | | |
| --- | --- | --- | --- | --- |
|  | **EPIDERMIS** | | **DERMIS** | |
| **GROUP** | **24h post UVR** | **72h post UVR** | **24h post UVR** | **72h post UVR** |
| 1—COX | 0.14 | 0.64 | **1.5x10^-4^** | 0.92 |
| 2—LOX | 9.2x10^-4^ | 0.038 | 0.32 | 0.14 |
| 3—CYP | 0.15 | 0.12 | 0.61 | 0.38 |
| 4—ENDO+NAE | 0.090 | 1.7x10^-3^ | 0.063 | **2.7x10^-4^** |
| 5—NAE | 0.059 | 2.9x10^-3^ | 0.087 | **1.3x10^-4^** |
| 6—HDHA | 2.0x10^-3^ | **1.3x10^-4^** | 8.9x10^-3^ | 0.013 |
| 7—HEPE | **2.8x10^-4^** | 5.4x10^-3^ | 0.27 | 0.015 |
| 8—HETE | 0.34 | 0.90 | 0.18 | 0.20 |
|  | **PC – downregulated** | | | |
|  | **EPIDERMIS** | | **DERMIS** | |
| **GROUP** | **24h post UVR** | **72h post UVR** | **24h post UVR** | **72h post UVR** |
| 1—COX | 0.41 | 0.014 | 0.93 | 4.4x10^-3^ |
| 2—LOX | 6.9x10^-3^ | 0.077 | 0.014 | 0.13 |
| 3—CYP | 0.014 | 0.095 | **4.6x10^-4^** | 0.18 |
| 4—ENDO+NAE | 0.059 | 0.45 | 0.16 | 0.70 |
| 5—NAE | 0.075 | 0.52 | 0.15 | 0.72 |
| 6—HDHA | 0.53 | 0.99 | 0.69 | 0.95 |
| 7—HEPE | 0.92 | 0.63 | 0.51 | 0.12 |
| 8—HETE | 0.18 | **3.5x10^-5^** | 0.30 | 0.10 |
